# DNAscan: personal computer compatible NGS analysis, annotation and visualisation

**DOI:** 10.1186/s12859-019-2791-8

**Published:** 2019-04-27

**Authors:** A. Iacoangeli, A. Al Khleifat, W. Sproviero, A. Shatunov, A. R. Jones, S. L. Morgan, A. Pittman, R. J. Dobson, S. J. Newhouse, A. Al-Chalabi

**Affiliations:** 1 Department of Biostatistics and Health Informatics, King’s College London, London, UK; 2 Department of Basic and Clinical Neuroscience, Maurice Wohl Clinical Neuroscience Institute, King’s College London, London, UK; 3 Department of Molecular Neuroscience, UCL, Institute of Neurology, London, UK; 4 Farr Institute of Health Informatics Research, UCL Institute of Health Informatics, University College London, London, UK; 5 National Institute for Health Research (NIHR) Biomedical Research Centre and Dementia Unit at South London and Maudsley NHS Foundation Trust and King’s College London, London, UK; 6 King’s College Hospital, Bessemer Road, London, SE5 9RS UK

**Keywords:** Bioinformatics, Variant calling, Viral detection, Repeat expansion, Structural variants, Annotation, Next generation sequencing

## Abstract

**Background:**

Next Generation Sequencing (NGS) is a commonly used technology for studying the genetic basis of biological processes and it underpins the aspirations of precision medicine. However, there are significant challenges when dealing with NGS data. Firstly, a huge number of bioinformatics tools for a wide range of uses exist, therefore it is challenging to design an analysis pipeline. Secondly, NGS analysis is computationally intensive, requiring expensive infrastructure, and many medical and research centres do not have adequate high performance computing facilities and cloud computing is not always an option due to privacy and ownership issues. Finally, the interpretation of the results is not trivial and most available pipelines lack the utilities to favour this crucial step.

**Results:**

We have therefore developed a fast and efficient bioinformatics pipeline that allows for the analysis of DNA sequencing data, while requiring little computational effort and memory usage. DNAscan can analyse a whole exome sequencing sample in 1 h and a 40x whole genome sequencing sample in 13 h, on a midrange computer. The pipeline can look for single nucleotide variants, small indels, structural variants, repeat expansions and viral genetic material (or any other organism). Its results are annotated using a customisable variety of databases and are available for an on-the-fly visualisation with a local deployment of the gene.iobio platform. DNAscan is implemented in Python. Its code and documentation are available on GitHub: https://github.com/KHP-Informatics/DNAscan. Instructions for an easy and fast deployment with Docker and Singularity are also provided on GitHub.

**Conclusions:**

DNAscan is an extremely fast and computationally efficient pipeline for analysis, visualization and interpretation of NGS data. It is designed to provide a powerful and easy-to-use tool for applications in biomedical research and diagnostic medicine, at minimal computational cost. Its comprehensive approach will maximise the potential audience of users, bringing such analyses within the reach of non-specialist laboratories, and those from centres with limited funding available.

**Electronic supplementary material:**

The online version of this article (10.1186/s12859-019-2791-8) contains supplementary material, which is available to authorized users.

## Background

The generation of whole genome sequencing (WGS), whole exome sequencing (WES) or targeted gene panels, is now standard practice in biomedical research. On a large scale, international sequencing consortia study the genetic landscape of thousands of individuals. On an individual scale, sequencing data are also used in diagnostic medicine and so called Precision Medicine [1, 2], with the aim to tailor medical treatments to patient genetics. There are several practical challenges when processing next generation sequencing (NGS) data. For example, WGS data for one sample produced on the Illumina Hiseq X, one of the most popular sequencers, is about 100 gigabytes, allowing for a high depth of sequencing (average 40x), when stored using lossless compressed formats such as fastq.gz. Such large files are not easy to handle for the average non-specialised scientist or lab, since they require sophisticated tools, bioinformatics skills and high performance computing clusters for analysis. While such facilities are available in specialist, well-resourced centres in wealthy countries, they are not readily accessible in other settings. Cloud computing provides a solution for the computing aspect of the challenge, but not the cost or the specialist skills needed. Furthermore, privacy requirements, ownership policies, and lack of an adequate internet infrastructure can make their use impractical.

A further significant issue is the large number of bioinformatics tools available for NGS analysis. Omictools [3], a web database where most available tools are listed and reviewed, lists over 7000 bioinformatics NGS tools, and new ones are frequently released. Among these more than 100 analysis pipelines are listed, most of which do not cover the whole data analysis, annotation and visualisation process and are computationally more intensive. For example, SpeedSeq [4] and GATK Best Practise Workflow [5] (GATK BPW) are two of the most popular. While these pipelines guarantee a very high genotyping quality, their use requires high-performance computing facilities and specialized expertise. What is needed therefore is a single pipeline, able to be deployed by someone without training in bioinformatics, and able to run on readily available computing equipment, easily accessible to non-specialist labs in any part of the world.

Here we describe DNAscan, an extremely fast, accurate and computationally light bioinformatics pipeline for the analysis, annotation and visualisation of DNA next generation (short-reads) sequencing data. DNAscan is designed to provide a powerful and easy-to-use tool for applications in biomedical research and diagnostic medicine, at minimal computational cost. The pipeline can analyse 40x WGS data in 13 h using 4 threads and 16 Gb RAM and WES data in 1 h using 4 threads and 10.5 Gb of RAM, and detect SNVs, small indels, structural variants, repeat expansions and viral genetic material (or that of any other microbe, e.g. bacteria and fungi). Results are annotated using a variety of databases and made available for a local deployment of the gene.iobio platform for an on-the-fly visualisation. Additionally, user-friendly quality control and results reports are generated.

## Material and methods

### Pipeline description

**Fig. 1:**
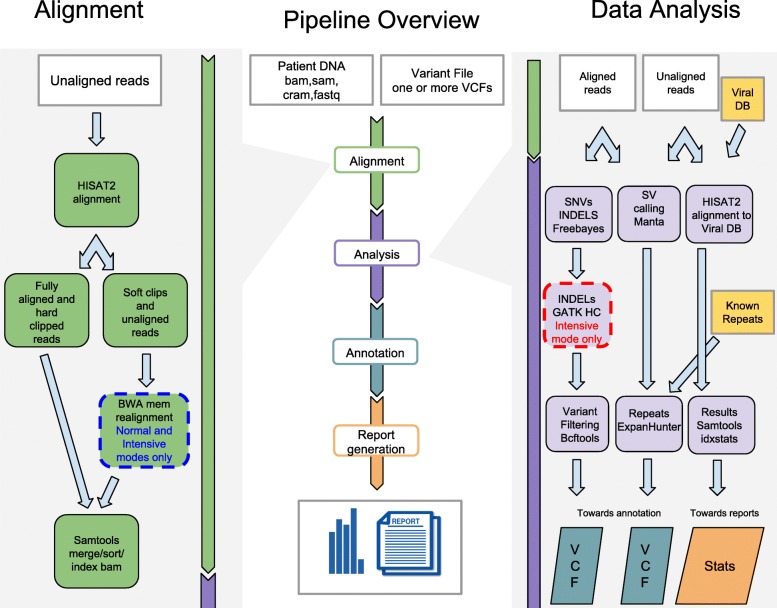
Pipeline overview. Central panel: DNAscan accepts sequencing data, and optionally variant files. The pipeline firstly performs an alignment step (details in the left panel), followed by a customisable data analysis protocol (details in the right panel). Finally, results are annotated and user-friendly QC and result reports are generated. The annotation step uses Annovar to enrich the results with functional information from external databases. Right panel: detailed description of the post alignment analysis pipeline. Aligned reads are used by the variant calling pipeline (Freebayes + GATK HC); both aligned and unaligned reads are used by Manta and ExpensionHunter (for which repeat description files have to be provided) to look for structural variants. The unaligned reads are mapped to a database of known viral genomes (NCBI database) to screen for their DNA in the input sequencing data. Left panel: Alignment stage description. Raw reads are aligned with HISAT2. Resulting soft-clipped and unaligned reads are realigned with BWA mem and then merged with the others using Samtools

The DNAscan pipeline consists of four stages: Alignment, Analysis, Annotation and Report generation, and can be run in three modes: Fast, Normal and Intensive, according to user requirements (Fig. 1 and Table 1). These modes have been designed to optimize computational effort without compromising performance for the type of genetic variant the user is testing (see mode recommendations in Table 2). The user can restrict the analysis to any sub-region of the human genome by proving either a region file in bed format, a list of gene names, or using the whole-exome option, reducing the processing time and generating region specific reports.

**Table 1:** Key tools used by DNAscan in the three modes

Stage	DNAscan mode		
	Fast	Normal	Intensive
Alignment	HISAT2	HISAT2 + BWA mem	HISAT2 + BWA mem
SNVs calling	Freebayes	Freebayes	Freebayes
Small indels calling	Freebayes	Freebayes	GATK HC

**Table 2:** DNAscan mode usage recommendations

Type of analysis	DNAscan mode		
	Fast	Normal	Intensive
SNVs	Yes	Yes	Yes
Small indels (< 50 bps)	No	No	Yes
Structural Variants	No	Yes	Yes
Repeat expansions	No	Yes	Yes
Non-human microbes	Yes	Yes	Yes

### Alignment

DNAscan accepts sequencing data in fastq.gz and as a Sequence Alignment Map (SAM) file (and its compressed version BAM). HISAT2 and BWA mem [6, 7] are used to map the reads to the reference genome (Fig. 1, left panel). This step is skipped if the user provides data in SAM or BAM formats. HISAT2 is a fast and sensitive alignment program for mapping next-generation sequencing reads to a reference genome. HISAT2 uses a new reference indexing scheme called a Hierarchical Graph FM index (HGFM) [8], thanks to which it can guarantee a high performance, comparable to state-of-the-art tools, in approximately one quarter of the time of BWA and Bowtie2 [9] (see Additional file 1).

Variant calling pipelines based on HISAT2 generally perform poorly on indels [10]. To address this issue, DNAscan uses BWA to realign soft-clipped and unaligned reads. This alignment refinement step is skipped if DNAscan is run in Fast mode.

Samblaster [11] is used to mark duplicates during the alignment step and Sambamba [12] to sort the aligned reads. Both the variant callers, Freebayes [13] and GATK Haplotype Caller (HC) [5] used in the following step, are duplicate-aware, meaning that they automatically ignore reads marked as duplicate. The user can optionally exclude it from the workflow according to the study design, e.g. when an intensive Polymerase Chain Reaction (PCR) amplification of small regions is required.

### Analysis

Various analyses are performed on the mapped sequencing data (Fig. 1, right panel): SNV and small indel calling is performed using Freebayes, whose reliability is well reported [14, 15]. However, taking advantage of the documented better performance of GATK HC in small indel calling, we decided to add a customised indel calling step to DNAscan, called Intensive mode. This step firstly extracts the genome positions for which an insertion or a deletion is present on the cigar of at least one read, and secondly calls indels using GATK HC on these selected positions. The reduced number of positions where this occurs allows for a targeted use of GATK HC, limiting the required computational effort and time. The resulting SNVs and small indel calls with genotype quality smaller then 20 and depth smaller than 10 are discarded. The user can customize these filters according to their needs (see GitHub [16] for details and a complete list of available filters).

Two Illumina developed tools, Manta [17] and Expansion Hunter [18] are used for detecting medium and large structural variants (> 50 bp) including insertions, deletions, translocations, duplications and known repeat expansions. These tools are optimised for high speed and can analyse a 40x WGS sample in about one hour using 4 threads, maintaining a very high performance.

DNAscan also has options to scan the sequencing data for microbial genetic material. It performs a computational subtraction of human host sequences to identify sequences of infectious agents including viruses, bacteria or fungi, by aligning the non-human or unaligned reads to the whole NCBI database [19–21] of known viral, bacterial or any custom set of microbial genomes and reporting the number of reads aligned to each non-human genome, its length and the number of bases covered by at least one read.

### Annotation

Variant calls are then annotated using Annovar [22]. The annotation includes the use of databases such as ClinVar [23], Exac [24], dbSNP [25] and dbNSFP [26] (more information about how to customise the annotation, e.g. by selecting alternative databases and/or focusing on specific genome regions, are available on GitHub).

### Reports and visualization utilities

DNAscan produces a wide set of quality control (QC) and result reports and provides utilities for visualisation and interpretation of the results.

MultiQC [27] is used to wrap up and visualise QC results. FastQC [28], Samtools [29] and Bcftools [30] are used to perform QC on the sequencing data, its alignment and the called variants. An example is available on GitHub [31]. A tab delimited file including all variants found within the selected region is also generated [32]. This report would include all annotations performed by Annovar [22] in a format that is easy to handle with any Excel-like software by users of all levels of expertise.

Three iobio services (bam.iobio, vcf.iobio and gene.iobio) are locally provided with the pipeline allowing for the visualisation of the alignment file [33], the called variants [34] and for a gene based visualisation and interpretation of the results [35].

### DNAscan benchmark

Benchmarking every DNAscan component is not needed since a range of literature is available [14, 15, 17, 36, 37]. However, to our knowledge, none exists assessing HISAT2 [8] (the short-read mapper used by the pipeline) either for DNA read mapping or as part of DNA variant calling pipelines. In this manuscript, we both assess the performance of HISAT2 with BWA and Bowtie2 [9] mapping 1.25 billion WGS reads sequenced with the Illumina Hiseq X and 150 million simulated reads (see Additional file 1), and compare our SNV/indel calling pipeline in Fast, Normal and Intensive modes with the GATK BPW [5] and SpeedSeq [4] over the whole exome sequencing of NA12878. Illumina platinum calls are used as true positives [38].

We also show how DNAscan represents a powerful tool for medical and scientific use by analysing real DNA sequence data from two patients affected by Amyotrophic Lateral Sclerosis (ALS) and of HIV infected human cells. For the ALS patients we use both a gene panel of 10 ALS-related genes, whose feasibility for diagnostic medicine has been previously investigated [2], sequenced with the Illumina Miseq platform, and the WGS data from the Project MinE sequencing dataset [39]. DNAscan was used to look for SNVs, small indels, structural variants, and known repeat expansions. The WGS of an HIV infected human cell sample [40] was used to test DNAscan for virus detection.

### Variant calling assessment

To assess the performance of DNAscan in calling SNVs and indels, we used the Illumina Genome Analyzer II whole exome sequencing of NA12878. Illumina platinum calls [38] were used as true positives.

GATK BPW calls were generated using default parameters and following the indications on the GATK website [41] for germline SNVs and indels calling. These include the pre-processing and variant discovery steps for single sample, i.e. skipping the Merge and Join Genotype steps.

SpeedSeq calls were generated running the “align” and “var” commands as described on GitHub [42]. RTG Tools [43] (“vcfeval” command) was used to evaluate the calls. F-measure, Precision and Sensitivity are defined as in the following: $Precision=\frac{T_p\ }{T_p+{F}_p}$, $Sensitivity=\frac{T_p\ }{T_p+{F}_n}$ and $F- measure=2\times \frac{Precision\times Sensitivity}{Precision+ Sensitivity}$, where *T*_*p*_ is true positives, *F*_*p*_ false positives and *F*_*n*_ false negatives.

### ALS Miseq and whole genome sequencing test cases

Using DNAscan in Fast mode, we analysed real DNA sequence data from two ALS patients (case A and case B). Case A carries a non-synonymous mutation in the *FUS* gene [44] (variant C1561T, amino acid change R521C, variant dbSNP id rs121909670 [45]) known to be a cause of ALS (ClinVar id RCV000017611.25). A panel of 10 ALS related genes was sequenced with the Illumina Miseq platform for case A. The Miseq gene panel was designed and tested for diagnostic purposes [2]. For these 10 genes (*BSCL2*, *CEP112*, *FUS*, *MATR3*, *OPTN*, *SOD1*, *SPG11*, *TARDBP*, *UBQLN2*, and *VCP*), the full exon set was sequenced, generating over 825,000,222-base-long paired reads. DNAscan was used to call SNVs, indels, and structural variants on case A.

Case B has a confirmed *C9orf72* expansion mutation on one allele, also known to be causative of ALS [46]. This expansion mutation is thousands of repeats long. and 40x WGS data was generated with the Illumina Hiseq X for case B. The WGS sample (paired reads, read length = 150, average coverage depth = 40) was sequenced as part of the Project MinE sequencing dataset [39]. For this sample we ran DNAscan on the whole genome. However, both for practical reasons and to simulate a specific medical diagnostic interest, we focused our analysis report on the 126 ALS related genes reported on the ALSoD webserver [47] and also looked for the *C9orf72* repeat.

For both samples, we also reported variants linked to frontotemporal dementia, which is a neurodegenerative disease that causes neuronal loss, predominantly involving the frontal or temporal lobes, with a genetic and clinical overlap with ALS [48, 49].

### *C9orf72* repeat primed PCR

Pathological *C9orf72* gene hexanucleotide repeat expansions were determined using repeat primed PCR (RP-PCR), as previously described [50].

## Hardware

SpeedSeq was run on a single machine with 64 Gb of RAM and an Intel i7–670 processor. The other tests were performed using a machine with 16 Gb of RAM and an Intel i7–670 processor.

## Results

### Single nucleotide variant and small indels calling assessment

**Fig. 2:**
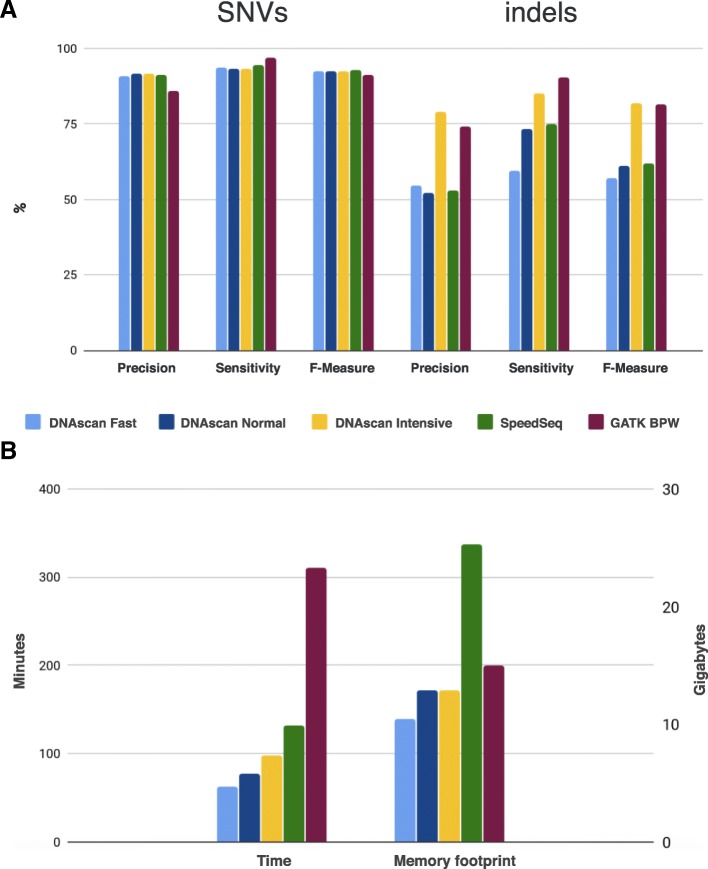
Variant calling assessment. Graph **a** shows the precision, sensitivity and F-measure of DNAscan in Fast, Normal and Intensive mode, SpeedSeq and GATK best practice workflow in calling SNVs and small indels over the whole exome sequencing of NA12878. Illumina platinum calls were used as true positives. The first three columns show the results for SNVs and the last three columns for indels. Graph **b** shows the time needed and the memory footprint for the same pipelines

To assess the performance of the DNAscan variant calling pipeline with GATK BPW and SpeedSeq, we used the exome of the well-studied NA12878 sample and the Illumina platinum calls as a gold standard (our set of true calls). The SpeedSeq pipeline uses BWA for alignment, Sambamba [12] and Samblaster [11] to sort reads and to remove duplicates, and Freebayes [13] for variant calling. Considering the overlap in the software used by DNAscan and SpeedSeq, assessing their performance is therefore of interest. Figure 2a shows the results from this test. DNAscan in Fast mode performs comparably with both the GATK BPW and the SpeedSeq on SNVs. Their F-measures (*F*_*m*_), a harmonic mean of precision and sensitivity defined in the Methods, are 0.92 (DNAScan), 0.91 (GATK BPW) and 0.93 (SpeedSeq).

In Normal mode DNAscan (*F*_*m*_ = 0.61) reaches an indel calling precision and sensitivity comparable to SpeedSeq (*F*_*m*_ = 0.62). The better performance of the Normal mode is driven by a major increase in sensitivity to 0.73 from 0.60. However, GATK BPW outperforms SpeedSeq on indels (GATK BPW *F*_*m*_ = 0.81). DNAscan, in Intensive mode, performs comparably to GATK BPW also on indels with an *F*_*m*_ of 0.82.

Figure 2b shows a comparison of the time needed by the tested pipelines and their memory usage. DNAscan in Fast mode completes the analysis in just 63 min while SpeedSeq takes over twice the time (132 min) and GATK BPW 5 times longer (310 min). DNAscan in both Normal and Intensive mode completes the analysis in a reasonable time (Normal 77 min, Intensive 98 min). DNAscan uses as little as 10.5 Gb RAM in Fast mode, and 12.9 Gb in Normal and Intensive mode, while GATK BPW uses 15 Gb and SpeedSeq over 25 Gb.

### Screening of ALS patients

For Case A, using the Miseq DNA gene panel, DNAscan detected 13 SNVs reported to be related to ALS and 4 to frontotemporal dementia on ClinVar, 6 non-synonymous variants and 6 variants with a deleteriousness CADD phred score [51] equal to or higher than 13, meaning that they are predicted to be in the top 5% most deleterious substitutions (Table 3). The known pathogenic *FUS* SNV rs121909670 was detected. No structural variants were found. The whole analysis was performed in ~ 30 min using 4 threads.

**Table 3:** Analysis of two ALS patients

	Case A	Case B
Analysis time (minutes)	30	460
Data size (MBs)	40	70,000
N. of ALS-related variants	13	33
N. of FTD-related variants	4	3
N. of non-synonymous variants	6	64
N. of variants with CADD> 13	6	748
N. long insertions	0	1
N. long deletions	0	3
N. Duplications	0	1
N. Inversions	0	0
*C9orf72* expansion	–	Yes
rs121909670	Yes	–

Case A was sequenced with targeted MiSeq ALS gene panel and carries a pathogenic non-synonymous mutation (rs21909670) in the *FUS* gene. Case B was whole-genome sequenced and carries a pathogenic *C9orf72* expansion

On the WGS data of Case B, for the selected 126 genes, DNAscan identified 33 SNVs reported to be related to ALS and 3 to frontotemporal dementia on ClinVar, 64 non-synonymous variants, 748 variants with a deleteriousness CADD phred score equal to or higher than 13, one 60-base-pair insertion, 3 over 100,000-base-pair long deletions and 1 tandem duplication. DNAscan was also able to detect the known *C9orf72* expansion (Table 3). The whole analysis was performed in ~ 8 h using 4 threads.

### Virus scanning

**Fig. 3:**
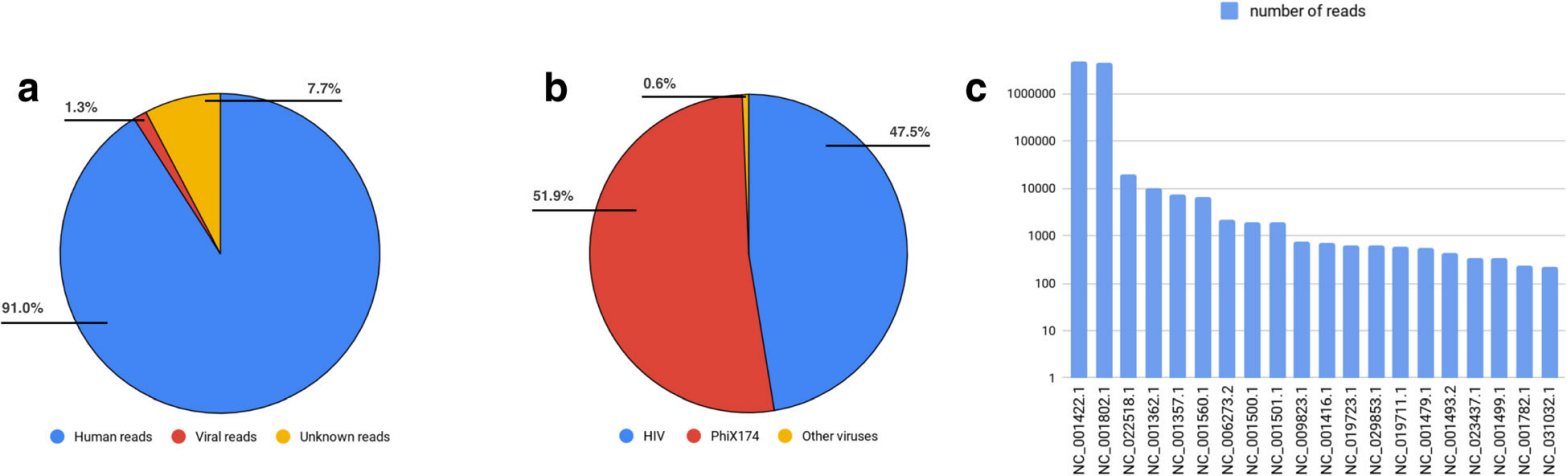
Identification of non-human reads Panel **a** shows the proportion of human reads (blue), viral reads (red) and unknown reads (yellow). Panel **b** shows the proportion for viral reads belonging to HIV (blue), PhiX174 (red) and to other viruses (yellow). Human reads are defined as reads which aligned to the human reference genome, viral reads as the reads which did not align to the human reference genome but aligned to at least one of the NCBI viral genomes and unknown reads as the reads which did not align either to the human or to any viral reference genomes. In panel **c** we plotted the numbers of aligned reads in logarithmic scale, for the 20 non-human microbe genomes to which the highest number of reads was aligned

We used DNAscan to detect the presence of viral genetic material in a whole genome sequencing sample of HIV infected human cells. The DNA sequencing data was produced using the Illumina Hiseq 2000 sequencer generating about 350 million 95-base length paired reads. Following the well-established approach of computational subtraction of human host sequences to identify sequences of infectious agents like viruses [52], the human reads (91%, Fig. 3a) were subtracted by mapping the sequencing data to the reference human genome using HISAT2. To screen our sequencing sample for the presence of known viral DNA, HISAT2 was then used to map the unmapped reads from the initial mapping phase of the pipeline (9%, Fig. 3a) to all the viral genomes available on the NCBI virus database.

Figure 3c shows a logarithmic representation of the number of reads aligned to the viral genomes in descending order for the 20 viral genomes to which the highest number of reads were mapped. They show both the presence of HIV DNA and bacterial DNA in our sample. Indeed, 4,412,255 reads mapped to the human immunodeficiency virus (NCBI id NC_001802.1) and only the Escherichia virus phiX174 (NCBI id NC_001422.1), a bacterial virus, presented a comparable number of reads (4,834,017 reads). This phage sequence is commonly found in Illumina sequencing protocols [53] probably because of transfer from gut microbes into blood.

Also, a smaller (3–4 orders of magnitude) number of reads belonging to other viruses were found. The disproportion between the presence of the first two hits (phiX174 and HIV) and the rest of the viruses is also shown in Fig. 3b. The complete results with the list of the whole set of viruses (120 viruses) for which at least one read was aligned can be found on GitHub [54]. The whole screening was performed by DNAscan using 4 threads in 2 h.

## Discussion and conclusion

DNAscan is an extremely fast, computationally efficient, easy to use pipeline for analysis, annotation and visualisation of next generation DNA sequencing data. It uses fast, but suboptimal tools to carry out first-line analysis, and optimal, but slower tools to refine the results. As a result, DNAscan is faster but not resource hungry, for example it is able to analyse 40x WGS data in 13 h and whole exome sequence data in one hour on a mid-range computer, performing as well as the widely used GATK BPW in terms of variant calling precision and sensitivity. Three different running modes, Fast, Normal and Intensive allow the pipeline to be tailored to specific needs while reducing time and RAM requirements compared to current standards, GATK BPW and SpeedSeq. It includes utilities for user friendly visualisation and interpretation of output. It is able to identify SNVs, structural variants, indels and expansion mutations, and favours use by non-specialists, and those with limited access to high performance computing facilities, for example, in less well-resourced countries or laboratories.

This comprehensive analysis approach aims to maximise the potential audience of users. However, NGS data can be used to investigate a very wide range of genetic variations which are impossible to enclose in only one analysis pipeline. DNAscan does not provide specific tools and protocols to detect whole classes of mutations, for example microsatellites, retrotransposons, novel or highly irregular repeat expansions and somatic variants. Moreover, it does not offer the adequate flexibility to allow different analysis approaches, for example consensus and meta variant calling that have been shown to be powerful strategies to detect SNVs and structural variants [55, 56] , or to analyse long-reads sequencing data such as PacBio and Nanopore [57, 58]. New implementations of DNAscan are already underway and will include new analysis protocols, including the detection of structural variants using long-read sequencing, the development of a webserver for an on-the-fly and a graphical user interface. However, the use of highly flexible and interactive analysis frameworks such as Seven-Bridges (www.sevenbridges.com), Galaxy [59], or ExScalibur [60] will remain a necessity for those users who need a higher degree of flexibility.

We also reported a few specific-use cases, such as the analysis of Miseq and WGS data of someone with ALS for diagnostic purposes, and the virus screening of HIV infected human cells. In the ALS test we showed how with MiSeq and Hiseq X WGS data, DNAscan detected a range of reported ALS-related variants in half an hour for the Miseq panel and 8 h for the WGS data (restricting the analysis to the 126 ALS genes), correctly reporting the presence of both the *C9orf72* expansion and the rs121909670 SNV. In the HIV test, DNAscan detected the expected viral presence by finding both the HIV virus and a phage commonly present in Illumina next generation DNA sequencing data.

Cloud computing and storage services offer practically unlimited computational power and storage. However, this has a cost, and optimisation, in particular for large scale sequencing projects, is of primary importance. Amazon Web Services (AWS) is one of the most popular cloud computing services. Performing the alignment, variant calling and annotation using DNAscan Fast mode on an EC2 instance [61] would cost about $2.41 (13 h of usage of a t2.xlarge machine with 4 CPUs). The same analysis using SpeedSeq would cost about $18.72 (10 h of usage of a h1.8xlarge machine with 32 CPUs). These prices do not take into account the storage, were updated on the 29th of April 2019 and take into consideration the cheapest machines available in the US East (Ohio) region matching the pipeline computational requirements proposed by the authors (4 CPUs and 16 Gb RAM for DNAscan and 32 CPUs and 128 Gb RAM for SpeedSeq [4]).

DNAscan is also available as a Docker and a Singularity image. These allow the user to quickly and reliably deploy the pipeline on any machine where either of these programmes is available. Singularity also allows for the deployment of the pipeline on environments for which the user does not have root permission. This could be particularly useful for users working on shared high performance computing facilities.

## Availability and requirements

Project name: DNAscan

Project home page: https://github.com/KHP-Informatics/DNAscan

Operating system(s): GNU/Linux based systems

Programming language: Python

Other requirements: https://github.com/KHP-Informatics/DNAscan#dependencies

License: MIT (https://github.com/KHP-Informatics/DNAscan/blob/master/LICENSE.txt)

Any restrictions to use by non-academics: no restrictions

## Additional file

Additional file 1: **Table S1.** Alignment assessment results. HISAT2, BWA and Bowtie2 were tested on 150 million simulated Illumina paired end human reads and 1.250 billion real Illumina paired end human reads. For the three aligners on the two dataset the table shows the time taken, their memory fingerprint and the percentage of aligned-one-or-more-times reads, aligned-only-once reads and properly pared. All tests were run using 4 threads. (DOCX 19 kb)

## Abbreviations

ALS: Amyotrophic lateral sclerosis
FTD: Frontotemporal dementia
GATK BPW: Genome analysis tool kit best practice workflow
GATK: Genome analysis tool kit
HC: Haplotype caller
HPC: High perfomance computing
INDEL: Insertion and deletion
NGS: Next generation sequencing
PCR: Polymerase chain reaction
QC: Quality control
SNV: Single nucleotide variant
SV: Structural variant
WES: Whole genome sequencing
WGS: Whole genome sequencing
