# Supplementary material for: DNAscan: personal computer compatible NGS analysis, annotation and visualisation
Source: BMC Bioinformatics. 2019 Apr 27;20:213. doi: 10.1186/s12859-019-2791-8 (PMC6487045; doi:10.1186/s12859-019-2791-8)
Supplement: Supplementary file 1 — Table S1. Alignment assessment results. HISAT2, BWA and Bowtie2 were tested on 150 million simulated Illumina paired end human reads and 1.250 billion real Illumina paired end human reads. For the three aligners on the two dataset the table shows the time taken, their memory fingerprint and the percentage of aligned-one-or-more-times reads, aligned-only-once reads and properly pared. All tests were run using 4 threads. (DOCX 19 kb) [file 12859_2019_2791_MOESM1_ESM.docx]

**Supplementary Material DNAscan**

**Authors**

A Iacoangeli^1,2^**^*^**, A Al Khleifat^2^, W Sproviero^2^, A Shatunov^2^, AR Jones^2^, SL Morgan^3^, A Pittman^3^, RJ Dobson^1,4,5^, SJ Newhouse^1,4,5^ and A Al-Chalabi^2,6^

**Affiliations**

^1^ Department of Biostatistics and Health Informatics, King’s College London, London, UK;

^2^ Maurice Wohl Clinical Neuroscience Institute, King’s College London, Department of Basic and Clinical Neuroscience, King’s College London, London, UK;

^3^ Department of Molecular Neuroscience, UCL, Institute of Neurology, London, UK;

^4^ Farr Institute of Health Informatics Research, UCL Institute of Health Informatics, University College London, London, UK;

^5^ National Institute for Health Research (NIHR) Biomedical Research Centre and Dementia Unit at South London and Maudsley NHS Foundation Trust and King’s College London London, UK.

^6^ King's College Hospital, Bessemer Road, London, SE5 9RS, UK.

***Correspondence** should be addressed to alfredo.iacoangeli@kcl.ac.uk

**The HISAT2 aligner assessment**

To assess the performance of the HISAT2 (1)aligner we used two datasets: 1.25 billion WGS reads of a human WGS DNA sample sequenced with an Illumina Hiseq 2000 (see Methods), and 150 million simulated human reads.

The simulated human reads were generated using pIRS (2) with default parameters and hg19.

In this assessment we took into consideration the memory footprint (RAM), the time needed to complete the alignment, percentage of reads mapped to the reference genome, and the percentage of uniquely mapped reads and properly paired reads. The performance of HISAT2 was compared with the BWA (3) aligner (mem algorithm (4)) and Bowtie2 (5), which are two of the most widely used small reads aligners. Supplementary table 1 shows the results from this test.

On this real dataset, HISAT2 uses 4.2 gigabytes of RAM, slightly larger than Bowtie2 (3.8 Gb RAM), while BWA has the biggest memory usage (9.1 Gb RAM). In terms of speed, HISAT2 completes the mapping in 4 hours while both the other aligners take about 4-5 times longer (19 hours 27 minutes for BWA and 17 hours 50 minutes for Bowtie2). In terms of percentage of uniquely mapped reads, HISAT2 closely compares with BWA (86.17% and 86.80% respectively) outperforming Bowtie2 (75.14%).

On the simulated dataset, all aligners perform well, however HISAT2 is over 4 times faster than the others, although uniquely aligning slightly fewer reads (97.75% versus BWA-MEM 100%).

These results highlight how HISAT2 performs comparably to BWA and Bowtie2 while keeping a low memory footprint (4.2 Gb RAM) and the highest speed (over 4 times faster than the other aligners on the real dataset).

|  | Simulated reads | | | Real reads | | |
| --- | --- | --- | --- | --- | --- | --- |
|  | HISAT2 | BWA-MEM | BOWTIE2 | HISAT2 | BWA-MEM | BOWTIE2 |
| Number of reads (Millions | 150 | 150 | 150 | 1250 | 1250 | 1250 |
| Time (minutes) | 32 | 130 | 115 | 245 | 1167 | 1070 |
| Memory fingerprint (Gigabytes) | 4.2 | 6.6 | 3.8 | 4.2 | 9.1 | 3.8 |
| Aligned reads (%) | 99.82 | 100 | 99.98 | 90.14 | 96.22 | 93.89 |
| Uniquely aligned reads (%) | 97.7 | 100 | 99.98 | 86.17 | 86.80 | 75.14 |
| Properly paired reads (%) | 99.46 | 100 | 52.48 | 85.61 | 95.60 | 62.55 |

**Supplemetary Table 1. Alignment assessment results.** HISAT2, BWA and Bowtie2 were tested on 150 million simulated Illumina paired end human reads and 1.250 billion real Illumina paired end human reads. For the three aligners on the two dataset the table shows the time taken, their memory fingerprint and the percentage of aligned-one-or-more-times reads, aligned-only-once reads and properly pared. All tests were run using 4 threads.

**References**

1. Kim, D., Langmead, B. and Salzberg, S.L. (2015) HISAT: a fast spliced aligner with low memory requirements. *Nat Methods*, **12**, 357-360.

2. Hu, X., Yuan, J., Shi, Y., Lu, J., Liu, B., Li, Z., Chen, Y., Mu, D., Zhang, H., Li, N. *et al.* (2012) pIRS: Profile-based Illumina pair-end reads simulator. *Bioinformatics*, **28**, 1533-1535.

3. Zaharia, M., Bolosky, W.J., Curtis, K., Fox, A., Patterson, D., Shenker, S., Stoica, I., Karp, R.M. and Sittler, T. (2011) Faster and More Accurate Sequence Alignment with SNAP. *ArXiv e-prints*.

4. Li, H. (2013) Aligning sequence reads, clone sequences and assembly contigs with BWA-MEM. *ArXiv e-prints*.

5. Langmead, B. and Salzberg, S.L. (2012) Fast gapped-read alignment with Bowtie 2. *Nat Methods*, **9**, 357-359.
